# Supplementary material for: Long-Term Trajectory and Risk Factors of Healthcare Workers’ Mental Health during COVID-19 Pandemic: A 24 Month Longitudinal Cohort Study
Source: Int J Environ Res Public Health. 2023 Mar 4;20(5):4586. doi: 10.3390/ijerph20054586 (PMC10002366; doi:10.3390/ijerph20054586)
Supplement: Supplementary file 1 [file ijerph-20-04586-s001.zip › ijerph-2187768-supplementary.pdf]

International Journal of
Environmental Research and Public Health

MDPI

Supplementary materials

Table S1. GHQ-12, IES-R, GAD-7 scores across subgroups: mean and standard deviation (sd) by gender, age, occupational role and Covid-19 area working experience. P values are referred to t-test and One-way ANOVA.

		GHQ-12		IES-R		GAD-7	
	N (%)	Time 1 mean (sd)	Time 2 mean (sd)	Time 1 mean (sd)	Time 2 mean (sd)	Time 1 mean (sd)	Time 2 mean (sd)
**Gender**							
Female	91 (29%)	3.85 (3.2)	2.15 (2.8)	23.3 (16.7)	13.5 (12.3)	6.93 (5.3)	5.33 (4.0)
Male	219 (71%)	3.48 (3.2)	1.78 (3.5)	18.8 (16.9)	12.4 (15.0)	5.43 (4.7)	4.27 (3.5)
		p=0.36	*p=0.26*	*p=0.03*	*p=0.52*	*p=0.01*	*p=0.02*
**Age group**							
20-29	36 (12%)	4.33 (3.2)	1.92 (2.5)	25.1 (17.6)	13.1 (11.3)	7.19 (4.8)	5.28 (3.9)
30-39	89 (29%)	4.06 (3.3)	2.28 (2.9)	22.8 (16.7)	15.1 (16.0)	6.57 (5.0)	5.27 (4.2)
40-49	75 (24%)	3.36 (3.3)	2.20 (2.8)	19.3 (16.8)	13.3 (12.8)	6.20 (5.5)	5.23 (4.1)
≥ 50	110 (35%)	3.55 (3.0)	1.78 (2.7)	22.2 (16.8)	11.6 (11.2)	6.39 (5.3)	4.60 (3.6)
		p=0.31	*p=0.58*	*p=0.36*	*p=0.33*	*p=0.82*	*p=0.57*
**Occupational role**							
Physician	83 (27%)	3.37 (2.9)	1.84 (2.6)	17.4 (14.3)	11.7 (10.9)	5.59 (4.5)	4.35 (4.3)
Administrative staff	46 (15%)	2.65 (2.8)	1.50 (2.2)	17.5 (11.9)	9.56 (8.5)	5.09 (4.4)	4.41 (3.4)
Nurse	116 (37%)	4.52 (3.5)	2.36 (2.9)	26.8 (18.4)	15.4 (14.8)	7.52 (5.6)	5.49 (4.3)
Health assistant	15 (5%)	3.13 (3.4)	1.47 (2.0)	26.4 (20.5)	16.8 (16.9)	7.73 (6.1)	5.00 (3.8)
Others	50 (16%)	3.74 (2.9)	2.30 (3.0)	21.4 (17.1)	12.8 (13.9)	6.53 (5.2)	5.62 (4.1)
		p=0.007	*p=0.30*	*p<0.001*	*p=0.05*	*p=0.02*	*p=0.17*
**Covid-19 area**							
Never	140 (45%)	3.33 (2.9)	1.90 (2.6)	19.9 (15.0)	10.8 (11.1)	6.22 (5.0)	4.93 (3.9)
Only T1	130 (42%)	4.23 (2.8)	2.11 (2.9)	25.0 (18.8)	15.9 (14.9)	6.84 (6.8)	5.12 (2.2)
Only T2	11 (4%)	3.91 (3.4)	2.63 (2.7)	18.5 (15.7)	10.8 (7.5)	6.18 (5.3)	4.36 (4.0)
T1 and T2	29 (9%)	3.48 (3.3)	2.21 (2.9)	19.2 (15.1)	13.9 (13.9)	6.34 (5.0)	5.31 (3.8)
		p=0.13	*p=0.78*	*p=0.06*	*p=0.01*	*p=0.79*	*p=0.89*

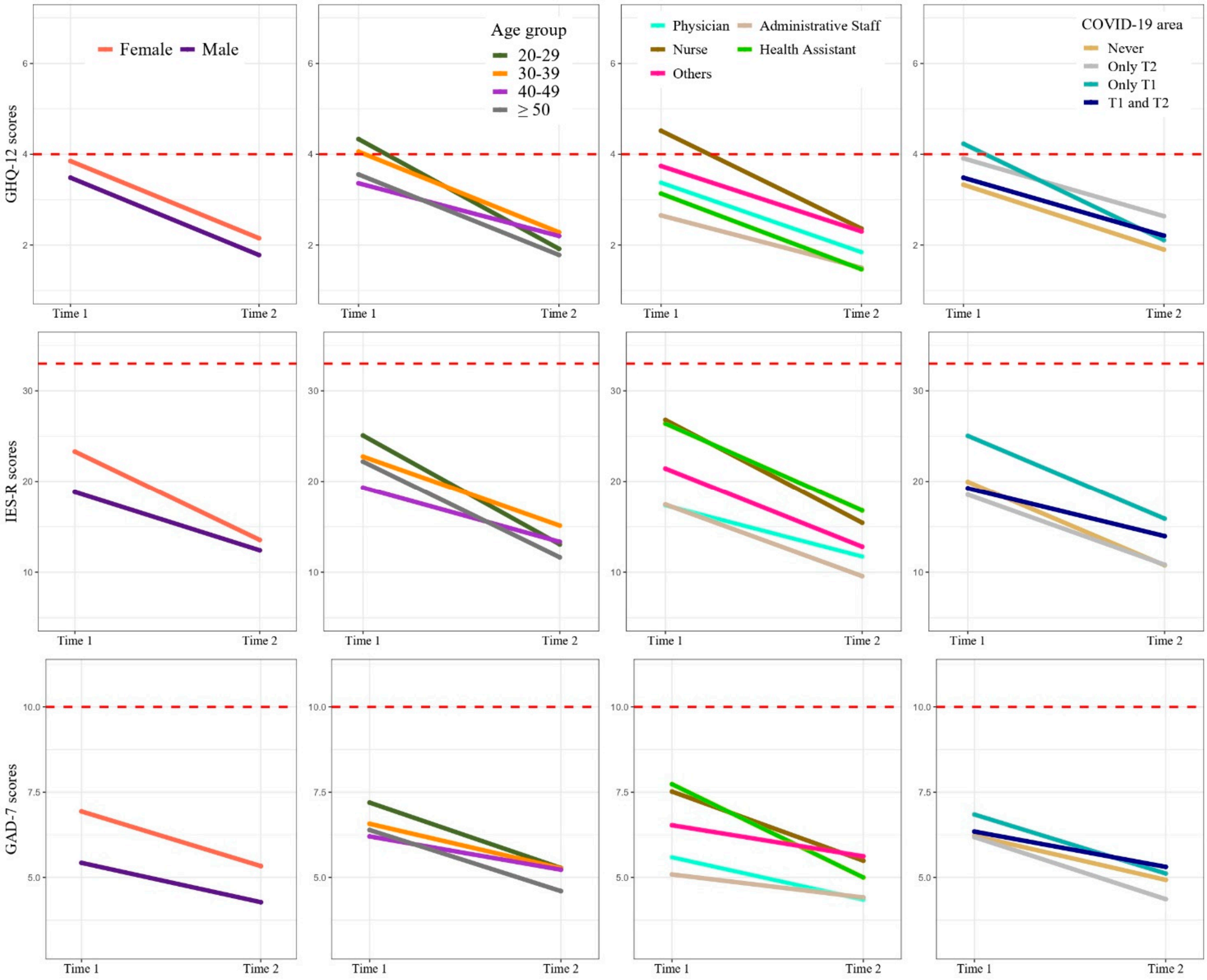

Figure S1. Mean scores trajectories from Time 1 to Time 2 by sub-groups (gender, age group, occupational role, Covid-19 area). Dotted red lines represent scales' cut-offs.
